# Sleep and circadian rhythms in adolescents with attempted suicide

**DOI:** 10.1038/s41598-024-57921-2

**Published:** 2024-04-09

**Authors:** Julie Rolling, Fabienne Ligier, Juliette Rabot, Patrice Bourgin, Eve Reynaud, Carmen M. Schroder

**Affiliations:** 1 Department of Child and Adolescent Psychiatry, Strasbourg University Hospitals, 1 Porte de l’Hôpital, BP 426, 67091 Strasbourg Cedex, Strasbourg, France; 2 Regional Center for Psychotraumatism Great East, Strasbourg University Hospital, Strasbourg, France; 3 CNRS UPR 3212, Institute for Cellular and Integrative Neurosciences, University of Strasbourg, Strasbourg, France; 4 University Center for Child and Adolescent Psychiatry, Nancy Psychotherapeutic Center, University of Lorraine, Nancy, France; 5 EA4360 APEMAC, MICS, University of Lorraine, Nancy, France; 6 Sleep Disorders Center, International Research Center for ChronoSomnology, Strasbourg University Hospitals, Strasbourg, France; 7 Centre de Recherche en Neurosciences de Lyon CRNL U1028, UMR5292, Université Claude Bernard Lyon 1, CNRS, INSERM, Forgetting, Bron, France

**Keywords:** Adolescence, Circadian rhythm, Sleep disturbance, Suicide attempt, Psychology, Human behaviour

## Abstract

Sleep and circadian rhythm disorders are very common in adolescents and have been linked to suicidal ideation. However, little is known about adolescent sleep before a suicide attempt (SA). The objectives of this study were to compare the sleep of adolescents aged 13 to 18 over a period of 4 weeks before a SA compared to a non-SA group, then to analyze the association between sleep, support social and well-being based on information from validated questionnaires. In 2015, 250 adolescents were included, 55 were recruited the day after a SA in French hospitals (before SA evaluations were retrospective). Logistic regression analyzes showed that during school days, bedtime was equivalent in both groups, but sleep onset latency was significantly longer in SA (86 min vs. 52 min, p = 0.016), and wake-up time was earlier (6 h 22 vs. 6 h 47, p = 0.002), resulting in a shorter total sleep time of 44 min (OR = 0.76, CI 95% [0.61–0.93]) the month preceding SA. Adolescents with longer sleep time performed better on perceived psychological well-being (p = 0.005), relationship with parents (p = 0.011) and school environment (p < 0.001). Results indicate a significant change in the quantity and quality of adolescents' subjective sleep in the 4 weeks preceding SA requiring objective measures to study the predictive properties of sleep in SA.

## Introduction

In France, death by suicide represents 16% of deaths in youth aged 15 to 24 years, making it the second cause of death in this age range^1^. Results from the SEYLE study, conducted in 11 European countries and carried out in 12,395 adolescent aged 14 to 16 years, show that the prevalence of declared suicide attempt (SA) was of 3% in males and 5% in females^2^. Amongst those who survive a suicide attempt, 12% relapse within the following 3 months^3^ and between 14 and 18%^4,5^ within the following 6 months, hence the utter importance of providing specific targeted therapeutic interventions for this population. Different risk factors of SA have been described in the literature, including family and personal history of SA, gender (female), psychiatric comorbidities^2,6,7^, lack of social support^8^ as well as sleep disturbances^9–13^.

Sleep and circadian rhythm disturbances are very common in adolescence, due to the developmental processes that occur at this stage of life^14^. Changes in sleep and rhythms lead to a progressive phase shift of sleep timing toward later hours, resulting in increasingly severe sleep deprivation in adolescents between 10 and 20 years of age, with 40.5% of self-reported sleep deprivation in 15-year-olds^15^. The impact of these changes is accentuated by the social environment, mostly school times, which do not allow adolescent to follow his or her later circadian preference or ‘chronotype’. This mismatch between the sleep–wake rhythms on ‘free days’ (when the adolescent can follow his or her usually delayed internal temporality) and those on school days defines the ‘social jetlag’^16^. As a results, the combination of insufficient sleep and circadian desynchronization exposes the adolescent to an increased risk of sleepiness during the day^15^, cognitive repercussions, emotional dysregulation and mood disorders ; Sivertsen and al. observed in 9338 adolescents an increase in inattention, hyperactivity, but also depression in subjects with a delayed sleep phase disorder^17^.

In addition, different studies have shown a specific association between sleep disturbances and suicidal ideation, but also suicidal risk, for adolescents in the general population. This association persists after adjustment for different factors (major depressive episode, post-traumatic stress, bullying at school, family and social environment, etc.), strongly suggesting that sleep and rhythm disturbances represent risk factors for suicide, independently of mental disorders 20). For example, studies in adolescents in the general population show that restricted total sleep time or nightmares are associated with increased suicidal ideation^18,19^. As such, Winsler and al. demonstrated in 27,939 adolescents aged 13 to 17 years that total sleep time reduced by one hour per night was associated with an increase in feelings of hopelessness, and an increase in suicidal ideation and suicide attempts^18^. Reduced sleep duration, late bedtime, and early morning awakenings have also been associated with increased suicidal risk in ethnically diverse adolescent populations^]^. Similarly, Nadorff and al. showed a persistent association between insomnia symptoms and suicidal ideation even after controlling for anxiety, depression, and post-traumatic symptoms (n = 583 students)^21^. Furthermore, daytime sleepiness appears to be a significant predictor of later suicidal behaviors in adolescents (n = 7072 adolescents of the general population)^22^. Finally, a social jet-lag of more than 2 h was associated with an increase in suicidal ideation, especially in girls^23^.

Overall, beyond a high prevalence of sleep disorders and associated sleep debt and sleep desynchronization in the general adolescent population and their link with psychological disorders, we note a strong association between sleep disturbances and suicidal ideation and risk in the general adolescent population, independently of underlying psychological or psychiatric disorders. These previous studies warranted further research in suicidal adolescents, in order to specify the importance of sleep disorders and circadian rhythm in the particular situational dynamics of the suicidal crisis, within this vulnerable adolescent population. The primary objective of our study was to describe subjective sleep and circadian rhythms in adolescents in the wake of an attempted suicide (SA), for the 4 weeks preceding the SA, compared to a non-SA group. The secondary objective was to analyze the association between sleep, circadian rhythm, and quality of life and social support in adolescent suicide attempters and non-suicidal adolescents.

## Material and methods

### Study population

SA adolescents were recruited as part of the MEDIADO study, a multi-center cross-sectional study designed to compare cellphone and social media use between adolescent who have made a suicide attempt (SA group) and a non-SA group of adolescents^24^. This study was approved by the ethical committee of Nancy University, France (‘Comité de Réflexion Éthique Nancéien Hospitalo-Universitaire’).

All inclusions in the SA group took place between February and August 2015 in the University Hospitals of Nancy and Strasbourg, located in Eastern France. Inclusion criteria for the SA group were to have been hospitalized in one of the inclusion centers up the days after a suicide attempt, to be between 13 and 18 years old, to have social security coverage and to have given informed consent to participate. Exclusion criteria were to not know how to read or write, and to not have access to either a cellphone or to an internet connection. The inclusion of the non-SA group was carried out in the same time period in two middle schools of the same region, including a rural and an urban establishment.

Overall, 58 adolescents were recruited in the SA group and 225 in the non-SA group.

### Measures

Data was collected in both groups using validated self-questionnaires.

#### Sleep and circadian rhythm measures

- Adolescents were asked the day after the suicide attempt to describe their sleep in the previous 4 weeks (before the suicide attempt for the SA group or before enrollment for the non-SA group), thus the data is of retrospective nature. The Munich Chronotype Questionnaire (MCTQ)^16^ was used to evaluate sleep and circadian rhythm parameters separately during school days (3 items) and free days (3 items). The MCTQ is largely used (completed by over 55 000 subjects aged 10 to 90 years^16^) and has been validated against the Horne–Østberg Morningness–Eveningness questionnaire (MEQ)^25,26^. The subjective sleep outcomes included of the MCTQ include Total Sleep Time (TST, the duration of perceived actual sleep, in hours) and Sleep Onset Latency (SOL, the subjective delay between bedtime and sleep onset, in minutes). The circadian rhythm parameters included the sleep schedule (bedtime, sleep onset time and wake up time, in decimal hours), chronotype and social jetlag. The chronotype defines the subject circadian preference which is a physiological trait and not a disorder in itself. Using the MCTQ’s quotation, it was determined based on the timing of mid-sleep on free days (midpoint between sleep onset and sleep offset), corrected for sleep-debt caused by school or work.
- The calculation is as follows^16,27^:
  $$MSF={Time\, of\, sleep\, onset}_{ on \,free\, days}+\frac{{TST}_{ on\, free\, days}}{2}$$
  $${MSF}_{SC}=MSF-0.5 \left({TST}_{on\, free \,days}-{TST}_{whole \,week}\right)$$
  with MSF the time of Mid-Sleep on Free days, MSFsc the time of Mid-Sleep on Free days Sleep-debt correct and TST the Total Sleep Time over the whole week, or only on free days as specified^27^.

Subjects with an MSFsc inferior or equal to 2.17 display a morning chronotype, those with an MSFsc superior or equal to 7.25 an evening type, and those in between have an intermediate chronotype^28^. MSFsc was used as a continuous variable for the analysis, and categories for descriptive purposes only. Social jetlag is defined as a mismatch between the circadian preference or chronotype of an individual (depending on his internal biological clock) and the rhythm of life depending on imposed external factors (school, work, leisure)^16^. Social Jetlag is assessed via the difference between the midpoint of sleep on school days compared to the one on free days^29^. If the midpoint of sleep differs by 2 h or more, a subject is considered in social jetlag^16^. Lastly, we calculated a difference in total sleep time between free days and school days (TST Δ, in hours). To describe the population, sleep debt was defined as a TST Δ > 2 h.

### Measure of perceived quality of life, health and social support

The perceived social support was evaluated from 12 items of the Multidimensional Scale of Perceived Social Support (= MSPSS). The MSPSS is a self-report with Likert scale (5 levels). There are 3 subscales, each addressing a different source of support: from family members, from friends, and from significant others. It has been already used in different countries^30,31^ and has been translated and validated in French^32^. This scale is well adapted to the adolescent population. It’s psychometric properties reveal a good internal coherence, a good reliability of the construct and a good test–retest validity^33,34^.

The perceived quality of life and health have been explored by the ‘Vécu et Santé Perçue de l’Adolescent’ (VSP-A) and by the Kidscreen-27.

The VSP-A is a French health-related quality of life questionnaire developed specifically for adolescents. It is a self-report questionnaire composed of 42 items exploring 7 dimensions: psychological well-being, energy/vitality, friends, parents, leisure, future and school. This scale assesses components of social support. It has a good internal coherence (alpha de Cronbach: 0.74 à 0.91), as well as a good content and construct validity^35,36^.

The Kidscreen-27 is a self-report quality of life questionnaire which has been validated in 12 European countries. It contains 27 items divided into 5 subscales: physical well-being, psychological well-being, parent’s relations and autonomy, social support and peers and school environment. It has a very good internal coherence with an alpha Cronbach superior or equal to 0.80 and a good discriminating power (Ferguson’s δ: 0.96 to 0.98, which is superior to the acceptable cut-off set to 0.70^37,38^).

### Analyses

Statistical analyses were computed using R® software. For primary study objectives, we first conducted bivariate analyses to compare differences in the SA group versus the non-SA group regarding their sleep and circadian rhythms, using Chi 2 for qualitative variables and Mann–Whitney U-test for quantitative variables, since the data was not normally distributed. We repeated the analyses adjusting on age and gender, using logistic regression, with the group as the dependent variable. Total sleep time was also studied as a categorical variable (≤ 6 h, 7 h, 8 h, 9 h and ≥ 10 h) as previous studies reported a non-linear association with suicide attempt. To answer to our secondary objective, we then conducted linear regression analyses adjusted on age and gender to analyses the association between total sleep time on school days and sleep latency on school days, with psychometric variables. We investigated the difference in these associations between the SA and the non-SA group by testing the group as an interaction term in the regression analyses. Statistical significance was set to α inferior to 0.05for all tests, and interactions were reported starting at p < 0.15.

Due to a significant difference between the groups regarding sex ratio and age, we additionally conducted sensitivity analyses comparing the SA group to a sample matched by age and sex, which we named control group. Two subjects within the SA group could not be age-match with non-SA participants and were thus excluded, reducing the SA group to 53 subjects in the sensitivity analyses.

### Ethics declarations

This study was approved by the ethical committee of Nancy University, France (‘Comité de Réflexion Éthique Nancéien Hospitalo-Universitaire’).

### Guidelines and regulations

The authors confirm that all research was conducted in accordance with the relevant regulations. Informed consent was obtained from all participants and their legal guardians. This research was conducted in accordance with the Declaration of Helsinki.

## Results

### Population description

Amongst teenagers recruited in the MEDIADO study, 195 subjects in the non-SA group (78%) and 55 subjects in the SA group (95%) answered to at least one of the questions regarding sleep and circadian rhythms and were thus included in the analyses. Included and non-included subjects did not differ according to age with respectively a mean age (SD) of 14.2 (1.1) years versus 13.9 (1.2) years (p = 0.30). However, they did differ according to gender, since 15.7% (N = 18) of the boys did not answer to any questions regarding sleep compared to 6.9% (N = 18) for girls; boys were thus excluded in greater proportions (p = 0.018). The majority of adolescents (95%) who attempted suicide self-assessed their sleep and quality of life (filling out self-questionnaires) within 48 h of the suicide attempt.

The demographic characteristics as well as the clinical sleep parameters of the included subjects are described in Tables 1 and 2. Amongst the recruited subjects, questionnaire filling rate was superior to 84.4% for all considered variables, with a mean of 93.6%.

**Table 1:** Primary analyzes of demographic and clinical sleep parameters (% (N) or mean (SD)).

	Total sample N = 250	SA group N = 55	Non-SA group N = 195	*p*
Demographic				
Age (years)	14.16 (1.09)	14.59 (1.54)	14.04 (0.9)	**0.001**
Gender (male)	39.27% (N = 97)	14.55% (N = 8)	46.35% (N = 89)	**< 0.001**
Previous suicide attempt (yes)	12.80% (N = 32)	56.36% (N = 31)	0.51% (N = 1)	**< 0.001**
Clinical sleep parameters				
Sleep onset latency > 30 min	11.07% (N = 27)	21.57% (N = 11)	8.29% (N = 16)	**0.007**
Chronotype^a^				
Morning	1.40% (N = 3)	0.00% (N = 0)	1.72% (N = 3)	NA
Neutral	85.05% (N = 182)	90% (N = 36)	83.91% (N = 146)	NA
Evening	13.55% (N = 29)	10% (N = 4)	14.37% (N = 25)	NA
Sleep debt^b^	36.45% (N = 78)	47.50% (N = 19)	33.91% (N = 59)	0.107
Social jet lag (decimal hours)^c^	2.89 (1.55)	2.80 (1.60)	2.91 (1.55)	0.771

Significant values are in bold.

SA group: adolescents after attempted suicide.

^a^Morning chronotype: Middle Sleep on Free days corrected for sleep debt (MSFsc) ≤ 2.17. Neutral chronotype: MSFsc [2.17–7.25]. Evening chronotype: MSFsc > 7.25.

^b^Sleep debt: Difference in the total sleep time on free days versus school days of > 2 h.

^c^Social jet lag: Difference in the midpoint of sleep on free days versus school days, in decimal hours.

**Table 2:** Sensitivity analyzes of demographic and clinical sleep parameters (% (N) or mean (SD)).

	SA group N = 53	Control group N = 53	*p*
Demographic			
Age (years)	14.54 (1.53)	14.36 (1.08)	0.319^a^
Gender (male)	15.09% (N = 8)	13.21% (N = 7)	0.781^a^
Previous suicide attempt (yes)	56.6% (N = 30)	1.89% (N = 1)	**< 0.001**
Clinical sleep parameters			
Sleep onset latency > 30 min	21.57% (N = 11)	7.55% (N = 4)	**0.042**
Chronotype^b^			0.372
Morning	0% (N = 0)	3.92% (N = 2)	NA
Neutral	90% (N = 36)	82.35% (N = 42)	NA
Evening	10% (N = 4)	13.73% (N = 7)	NA
Sleep debt^c^	47.5% (N = 19)	33.33% (N = 17)	0.170
Social jet lag (decimal hours)^d^	2.80 (1.66)	2.80 (1.60)	0.819

Significant values are in bold.

SA group: adolescents after attempted suicide.

^a^Matching variables.

^b^Morning chronotype: Middle Sleep on Free days corrected for sleep debt (MSFsc) ≤ 2.17. Neutral chronotype: MSFsc [2.17–7.25]. Evening chronotype: MSFsc > 7.25.

^c^Sleep debt: Difference in the total sleep time on free days versus school days of > 2 h.

^d^Social jet lag: Difference in the midpoint of sleep on free days versus school days, in decimal hours.

Difference in sleep and circadian rhythms between the SA group and non-SA group.

**Figure 1:**
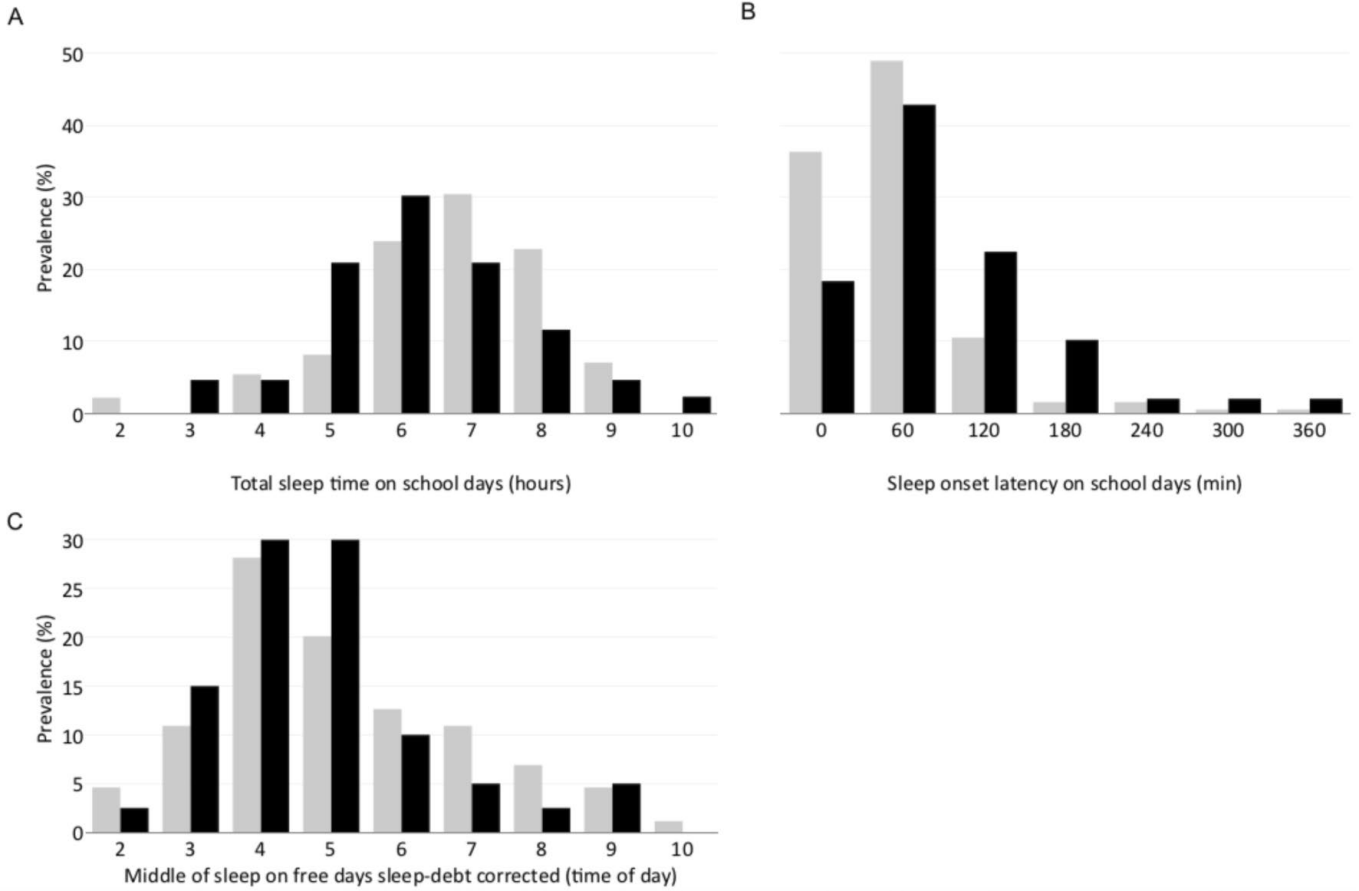
Sleep onset latency, sleep duration and sleep phase in adolescents who have attempted suicide (SA) compared to controls. Distribution of (**A**) hours of total sleep time on school days, (**B**) sleep onset latency on school days, and (**C**) midpoint of sleep on free days corrected for sleep debt, in adolescents who have attempted suicide (SA, black bars) non-SA group (grey bars). The data is represented as categories for illustrative purposes, it is used as continuous variables for the analysis.

Sleep and circadian rhythms in both groups are described in Tables 3 and 4, and distribution differences are illustrated in Fig. 1 for TST, SOL and MSFsc. According to the bivariate analyses, almost all sleep parameters on school days differed between groups. Adolescents in the SA group had similar bed times on school days than the non-SA group, but fell asleep 35 min later (p = 0.023) and woke-up 25 min earlier (p < 0.01), thus sleep onset latency was significantly longer (+ 34 min, p < 0.001), and total sleep time significantly shorter (− 44 min, p = 0.004). On the contrary, none of the sleep parameters differed on free days. However, the difference in total sleep time between free days and school days (TST Δ) was also bigger in the SA group (TST Δ of 2 h and 35 min in the SA group compared to 1 h and 44 min in non-SA group, p = 0.011), but no differences were observed regarding MSFsc chronotype. These associations remained significant when adjusting for age and sex, as reported in Tables 3 and 4. Of note, the sensitivity analyses, based on age and sex matched samples, produced the same results, with the exception of sleep onset latency on school days, where the 25 min difference between the SA and the control group was not statistically significant.

**Table 3:** Primary analyzes of sleep and circadian rhythm in the suicide attempt group and non-SA group.

	SA group N = 55	Non-SA group N = 195	OR [CI_95%_]	*p*
Sleep on school days				
Bed time (time of day)	22:13 (1:21)	22:15 (1:16)	1.11 [0.86–1.44]	0.401
Time of sleep onset (time of day)	23:40 (1:44)	23:05 (1:27)	1.32 [1.07–1.64]	**0.009**
Wake up time (time of day)	06:22 (0:50)	06:47 (0:40)	0.44 [0.26–0.74]	**0.002**
Sleep onset latency (hours)	01:26 (1:17)	00:52 (0:56)	1.44 [1.08–1.95]	**0.016**
Total sleep time (hours)	06:56 (1:05)	07:40 (1:03)	0.76 [0.61–0.93]	**0.010**
Sleep on free days				
Bed time (time of day)	23:53 (1:03)	24:11 (1:58)	0.98 [0.81–1.17]	0.813
Time of sleep onset (time of day)	25:21 (2:07)	25:04 (2:08)	1.11 [0.95–1.30]	0.186
Wake up time (time of day)	10:31 (2:11)	10:04 (1:58)	0.97 [0.80–1.16]	0.725
Sleep onset latency (hours)	01:20 (1:17)	00:56 (1:08)	1.27 [0.97–1.69]	0.090
Total sleep time (hours)	09:26 (1:45)	09:26 (1:47)	0.98 [0.80–1.20]	0.808
TST Δ^a^ (hours)	02:35 (2:01)	01:44 (1:49)	1.27 [1.03–1.58]	**0.026**
MSFsc^b^ chronotype (time of day)	04:50 (1:33)	05:01 (1:46)	0.87 [0.67–1.10]	0.255

Significant values are in bold.

^a^TST Δ: Difference in the total sleep time on free days and school days.

^b^MSFsc chronotype: Middle Sleep on Free days corrected for sleep debt.

Time of day and hours are reported in hours: minutes.

**Table 4:** Sensitivity analyzes of sleep and circadian rhythm in the suicide attempt group and control group, description and association adjusted on age and gender.

	SA group N = 53	Control group N = 53	OR [CI 95%]	*p*
Sleep on school days				
Bed time (time of day)	22:10 (1:19)	21:59 (1:05)	1.23 [0.87–1.75]	0.244
Time of sleep onset (time of day)	23:40 (1:44)	23:00 (1:31)	1.38 [1.06–1.83]	**0.020**
Wake up time (time of day)	6:22 (0:50)	6:44 (0:34)	0.43 [0.22–0.78]	**0.009**
Sleep onset latency (hours)	1:26 (1:17)	1:01 (1:14)	1.35 [0.97–1.96]	0.085
Total sleep time (hours)	6:56 (1:54)	7:43 (1:41)	0.73 [0.56–0.93]	**0.016**
Sleep on free days				
Bed time (time of day)	23:53 (1:30)	24:01 (2:07)	0.99 [0.79–1.23]	0.910
Time of sleep onset (time of day)	25:13 (1:56)	24:53 (2:18)	1.08 [0.89–1.31]	0.436
Wake up time (time of day)	10:25 (2:06)	10:28 (1:57)	0.94 [0.75–1.16]	0.574
Sleep onset latency (hours)	1:20 (1:17)	0:53 (1:00)	1.41 [0.96–2.23]	0.102
Total sleep time (hours)	9:28 (1:46)	9:32 (1:41)	0.93 [0.71–1.22]	0.602
TST Δ^a^ (hours)	2:35 (2:12)	1:50 (1:41)	1.31 [1.01–1.74]	**0.049**
MSFsc^b^ chronotype (time of day)	4:50 (1:33)	5:02 (1:53)	0.88 [0.66–1.14]	0.346

Significant values are in bold.

^a^TST Δ: Difference in the total sleep time on free days and school days.

^b^MSFsc chronotype: Middle Sleep on Free days corrected for sleep debt.

Time of day and hours are reported in hours: minutes.

We observed concurring results when studying total sleep time as a categorical variable. Adolescents with a total sleep time on school days of 6 h or less had an increased risk of belonging to the SA group compared to those who slept 9 h (OR = 4.57 [1.41–17.9], p = 0.017). Other total sleep time categories were not associated with significantly higher risk, individually. Figure 1 illustrates the patterns of a lower proportion of total sleep time (< 6 h) in suicidal adolescents and a higher proportion of sleep latency longer than 120 min.

### Association between sleep on school days and psychometric measures

The age and gender adjusted association between total sleep time and psychometric measures, namely MSPSS, VSP-A and Kidscreen-27, are reported in Table 5.

**Table 5:** Association between total sleep time on school days (minutes) with social support and wellbeing.

	β (SD) N = 250	*p*
MSPSS		
Significant other subscale	3.86 (4.94)	0.436
Family subscale	15.78 (4.06)	**< 0.001**
Friend subscale	3.67 (4.49)	0.414
Total score	12.37 (5.06)	**0.028**
VSP-A		
Psychological well-being	1.19 (0.39)	**0.003**
Energy/vitality	1.06 (0.34)	**0.002**
Friends	0.19 (0.29)	0.515
Parents	1.39 (0.25)	**< 0.001**
Leisure	− 0.45 (0.25)	0.077
School	0.57 (0.24)	**0.020**
Kidscreen-27		
Physical well-being	2.72 (1.66)	0.103
Psychological well-being	3.37 (1.18)	**0.005**
Parents relations & autonomy	2.94 (1.14)	**0.011**
Social support & peers	− 2.25 (1.54)	0.147
School environment	8.40 (1.71)	**< 0.001**
Score total	1.27 (0.42)	**0.003**

Significant values are in bold.

Linear regression analyses with total sleep time on school days as dependent variable, and a single social support or wellbeing as independent factor, adjusted on age and gender.

All family related subscales (MSPSS family subscale, VSP-A parent’s subscale and Kidscreen-27 parent relations and autonomy subscales) were positively associated with total sleep time on school days. Subjects with longer sleep on school days had higher scores on these scales, indicating a better perceived family social support, and relationship with parents.

There were also significant associations between total sleep time and all psychological well-being subscales, as well as all school related subscales, with longer total sleep time correlating with overall more positive well-being.

The VSP-A ‘energy/vitality’ subscale was positively associated with total sleep time for the full sample. However, the association between total sleep time and the Kidscreen-27 ‘physical well-being’ subscale showed an interaction by group (p of interaction = 0.102), where the ‘physical well-being’ subscale improved with longer sleep time only for non-SA subjects, with β = 4.50 (SD 1.74), p-value = 0.011, but was non-significant within the SA group (p = 0.656).

All results regarding friends, significant others and leisure activities did not show significant associations with total sleep time on school days.

## Discussion

This study found major differences between the sleep patterns of adolescent who attempted suicide and non-SA adolescents in the 4 weeks prior to the SA. Thus, total sleep time on school days was significantly lower by 44 min (p = 0.010) for the SA group than for the non-SA group, which was explained by a longer sleep latency and an earlier wake-up time, but not by a later bedtime. However, sleep on free days did not differ on any parameter between the two groups, with a bedtime close to midnight and a total sleep duration of about 9.5 h. The chronotype, defined by the sleep deficit-corrected midpoint of sleep on free-days (MSFsc), was also equivalent in the two groups. However, the difference in total sleep time on free days versus school days was greater for adolescents in the SA group (2:35 versus 1:44, *p* = 0.026), indicating that during the period surrounding the suicidal act, suicidal adolescents had a greater sleep debt accumulated during school days (due to the shorter total sleep time observed) and thus a need for more 'catch-up' sleep during weekends. The lack of difference between the two groups in chronotype and sleep on days off, combined with reduced sleep and earlier wake-up on school days in the SA group, demonstrates that the internal circadian timing system and the need for sleep are similar in the two populations, but that there is a significant mismatch between sleep need and actual sleep time in the SA group. Furthermore, subjects with longer total sleep time had higher perceived family social support, psychological well-being and also scored higher on the school-related subscales, but no association was found with the friends and peers’ subscales.

Our results are consistent with data from the literature showing that sleep disorders are associated with an increased risk of suicide attempts independent of psychopathology, particularly in individuals with sleep difficulties^39^, and also regarding the association between short sleep duration and suicide risk. Fitzgerald et al. showed that adolescents of the general population who slept 4 h or less on school nights had an increased risk of suicidal ideation (OR 2.7 CI 95% [2.0–3.7]), suicide attempt (OR 4.3 CI 95% [2.9–6.2]), and attempt requiring treatment (OR 6.5 CI 95% [3.2–13.0]) in the year prior to SA, indicating a higher severity of suicidality^9^. In contrast, Park et al. (2013) only found an association between sleep disturbance and suicidal ideation but not with suicide attempt^11^. One explanation for these differences may be that Park et al. did not differentiate between school days and weekend days^11^. Other studies have observed that the association between total sleep time and suicidality is not strictly dose-dependent, with an increased risk observed for both short sleepers and long sleepers along a U-shaped curve (≥ 10 h)^9,18,20^. This work is consistent with other work in larger cohorts of adults showing a similar distribution^40^. As such Guo et al. demonstrated, in 20,130 high school students in southeastern China, that short sleep (< 5 h) was positively associated with suicidal ideation (AOR = 2.28, 95% CI = 1.96–2.66) and suicide attempts (AOR = 3.20, 95% CI = 2.46–4.16), and that long sleep (> 9 h) was only significantly associated with suicide attempts (AOR = 2.47, 95% CI = 1.70–3.58)^10^. In the study by Winsler and al., the increase in suicidality for more than 10 h of sleep was greater for suicide ideation than for suicide attempts, whereas the reverse was observed by Guo and al. and Fitzgerald and al.^9,18,20^. In our study, adolescents who slept for 10 h or more only showed a trend toward a higher risk of belonging to the SA group. This could be explained either by the small number of adolescents sleeping 10 h in our sample (n = 16), the specificity of the study population whose suicide attempt was medically confirmed and required hospitalization, or the timing of the sleep duration assessment. Overall, these results highlight the highly significant link between sleep deficit and suicidal risk in adolescents, and the differential impact of sleep according to the severity of suicidality and the time of assessment, underscoring the need for studies specifically addressing sleep of adolescents hospitalized after a suicide attempt.

Recent studies have reported contradictory results concerning the association between total sleep time and suicide attempts in at-risk populations. Indeed, in the study by Kayowala and al (2015), which included adolescents hospitalized after a suicide attempt, short sleep duration was not a risk factor for SA^41^. The discrepancy with our results could be explained by the unspecified period of sleep assessment, as well as the lack of distinction between sleep patterns on school days and weekends. Moreover, our population consisted of adolescents, the majority of whom had a history of suicide attempts (56.4% for suicidal adolescents versus 0.51% for non-SA adolescents), which reflects a greater psychological vulnerability, explaining the concordance of our results with populations presenting more severe mental health disturbances^42^. As such, we can observe that sleep assessment in the context of psychological autopsy has highlighted a significant and temporal association between sleep problems (insomnia) and suicide in suicidal adolescents during the week preceding the suicidal act (n = 140 adolescents)^43^. Furthermore, our results confirm data from McGlinchey's study of 223 adolescents presenting to a community clinic for non-suicidal self-injurious and suicidal behaviors and mood disorders. Although their population is more heterogeneous, it appears that average insomnia and circadian reversal were both predictors of suicide attempts and that insomnia was significantly associated with suicidal ideation^42^.

Our results highlight a significant reduction in suicidal individuals' total sleep time during school time. As shown in the metanalysis of Astill (2012), sleep deprivation in young people is associated with deficits in complex cognitive functions (executive functions), which leads to an increase in behavioral problems (aggressive and destructive behaviors), but also to poorer school performance^44^. We know that poorer school performance decreases self-esteem and the feeling of exclusion, and that deficits of executive functions can also cause disinhibition and thus promote at-risk behaviors. In addition, sleep disturbance increases perceived isolation and lack of use of social support, which are protective factors for suicide^45,46^, whereas school bullying and cyber-bullying has been associated with suicidality in young people^47^.

Our results demonstrate that adolescents with longer total sleep time had higher perceived family social support and psychological well-being scores. Different studies conducted in the adult general population highlight a bidirectional relationship between social support quality and sleep quality^48,49^. Nevertheless, it is necessary to interpret these results with caution because, depending on the situation, social support can also involve social pressures that possibly alter sleep. In adolescents, recent studies show that social support increases sleep quality and decreases sleep deprivation^50^. Social support moderated the effects of academic stress on sleep, improving sleep quality and decreasing sleep reduction. These moderating effects were strongest during a period of high stress. Our results reflect the importance of a sufficient amount of sleep on social relationships and psychological well-being. These results are consistent with the findings of recent studies that show a bidirectional link between decline in the quality of social relationships with relatives and poor sleep quality with a robust association between social support and favorable sleep outcomes, both for adults^48,49,51^ and adolescents^52^. Beyond sleep duration and quality, we have also found that suicidal adolescents showed a tendency to have a greater phase shift of wake-up and bedtime schedules than non-SA adolescents. Phase shift can reach pathological levels, as in the case of delayed sleep-phase syndrome (DSPS). DSPS does not only affect cognitive functioning and mental health, but also social and family life and the well-being of the parents^52^. Thus, the impact of sleep on family and social ties must be taken into account, in particular given that family ties are protective factors with respect to suicide risk^46^. In this context, the impact of screen use on sleep must also be taken into account as the latest studies show consistently that screen use among adolescents, especially among heavy users of social media, further shifts circadian rhythms to later schedules, exacerbating sleep delays and thus sleep debt^53^. Thus, it would also be interesting to discuss the possibilities of adapting school schedules to the developmental changes (majority of evening chronotypes) associated with adolescence. Reduced sleep duration was associated with overall lower psychological well-being, which has been found in the general population literature^54^. Data from the literature assessing the link between sleep and physical state shows a combination of the two^55^. Our results are consistent with this for non-SA adolescents but not for suicidal individuals. We suggest that for our population the beneficial effect of sleep on physical activity would be masked by psychological distress at the time of the suicidal crisis.

Current research suggests that vulnerability to suicide is mediated by an underlying genetic predisposition that interacts with environmental and epigenetic factors throughout life (diathesis-stress model). According to this model, the set of life events experienced by an individual will modify neural circuits and adaptive capacities, making an individual more likely to adopt suicidal behavior^56^. In this context, sleep disorders could be involved in the suicidal act through different mechanisms, such as sleep deprivation related to sleep or circadian rhythm disorders (notably DSPS). Studies that have directly evaluated the relationship between sleep duration and emotion regulation^57^ as well as impulse control^58,59^ support the hypothesis that sleep deprivation has deleterious effects on inhibitory control mechanisms, emotionally^60^, but also cognitively (decision making and problem solving, judgment and discernment skills, etc.)^61,62^ including impulse control. These studies suggest that sleep deprivation can temporarily modify higher cognitive functions in certain social or environmental situations, via the prefrontal lobe^43^. Studies suggest a decrease in inhibitory connectivity between the medial prefrontal cortex (and the anterior cingulate cortex) with the amygdala^63–65^. The amygdala acts to modulate approach or withdrawal actions toward emotional stimuli in the environment. Sleep may facilitate stronger top-down prefrontal control of the amygdala, allowing for proper processing of emotional stimuli in the environment, whereas sleep loss would lead to increased impulsivity toward certain emotional stimuli^66^. In total, sleep deprivation seem to negate the top-down control that guides adaptive behaviors in crisis^67^. Thus, cognitive alterations (decision making and problem solving) and emotional dysregulation related to sleep deprivation are implicated in suicidal behaviors^40^ and self-harming behaviors^68,69^. Sleep deprivation may also lead to higher levels of impulsivity, increasing unplanned suicidal behaviors^70^, especially for vulnerable populations such as adolescents. Indeed, the prefrontal cortex continues to change until the age of 25 years, with the mismatch between the brain development of the limbic system and that of the prefrontal areas explaining greater impulsivity and emotional salience in suicidal youth^56^. In addition, the adolescent brain is considered to be the most sensitive to the effects of sleep loss because it undergoes significant metabolic changes between the waking and sleeping states. Thus, it is known that adolescents are more vulnerable to suicidal acting out because they have a higher level of impulsivity than at other ages of life, and that some youth may have vulnerabilities (history of childhood trauma) or psychological conditions (borderline personality disorder, conduct disorder…) that can further increase the level of impulsivity^71^. Finally, it is known that greater variability in nighttime sleep patterns has also been associated with decreased tendencies to select positive emotional situations, which may contribute to keeping subjects in cognitive distortions with poor coping strategies.

Neurobiological abnormalities such as dysfunctional serotonergic and dopaminergic systems, could also be involved in suicidal behavior, either linked to episodes of decompensation of psychological disorders or independently. The diathesis-stress model suggests that a deficit in serotonergic projections to the orbitofrontal cortex is involved in the susceptibility to suicidal behavior. Work carried out in suicidal patients suggests an association between REM sleep abnormalities and suicidal behavior, which could be linked to an alteration in serotonergic function^72^. Dysregulation of the serotoninergic system is particularly associated with violent suicides^73^. Finally, dysregulation of the serotonin transporter could contribute to mood disorders and possibly to the risk of suicide attempts by sensitizing the stress response system^74^. Overall, the link between sleep disorders and suicidal behaviors is strong but complex with bidirectional interactions, whether or not it is associated with a psychiatric disorder, or whether it is part of a framework of vulnerabilities (stress vulnerability model of suicide) reinforced by the developmental changes associated with adolescence. More transdisciplinary and integrative research is needed to better understand these interactions.

Although a growing body of literature shows that there is a (i) high prevalence of sleep disorders in populations of suicidal adolescents, as well as an (ii) association between sleep disorders and suicidal risk, both in the general population of adolescents and also in the population of suicidal adolescents, and this independently of a psychiatric diagnosis, our study is the first study to date to specifically compare sleep of adolescents in the month prior to a suicide attempt and evaluated just in the aftermath of the SA, with non-suicidal. Indeed, the literature has so far focused on assessing the quantity and quality of sleep in global mental health surveys^9,11,18,20^, without taking into account the time at which the SA took place. In addition, most of these public health studies are based on subjective reporting of a SA. In addition, previous studies of suicide risk factors have tended to focus on distal (e.g., adverse life events in early childhood) and time-invariant (e.g., demographic variables) factors^75^ or on psychiatric diagnoses that we know are less frequently associated with suicidality in adolescents compared to adults. Finally, contrary to others studies that explored the sleep of suicidal adolescents, we have distinguished sleep between school and weekend periods, in order to better characterize the sleep patterns of these adolescents^41–43^.

The first limitation of the study is its retrospective design, with adolescents asked to describe their sleep over the previous 4 weeks just in the wake of a SA. Adolescents who had recently attempted suicide might be more likely to describe poorer sleep quality and quantity, due to their psychological state at the time of the questionnaire. However, the similarity of sleep on free days between the two groups suggests that sleep difficulties are not systematically overreported in the SA group. The second limitation of the study concerns the method of data collection by self-report questionnaire because it does not allow objective and continuous data to be obtained. Indeed, subjective reports are not as accurate (overestimation) as objective measures^76^. Nevertheless, these reports are interesting because they reflect the youth's perception of his or her sleep. The third limitation of the study is the lack of sleep data for suicidal adolescents beyond 1 month before the suicide attempt. Indeed, the absence of previous data does not allow us to conclude whether sleep disorders are a marker of transient vulnerability or a pre-existing pathological state. Moreover, other dimensions of sleep could have been integrated (quality, continuity, regularity, satisfaction, etc.), because sleep duration requirements vary from one individual to another, as do other risk and protective factors for suicide. Therefore, our study cannot establish a causal relationship between sleep disturbance and suicide attempt. The fourth limitation is that the study included a relatively small number of subjects, which limits statistical power, especially for dichotomous variables such as 'clinical sleep parameters'. Similarly, a larger number of subjects would be needed to better understand the association between a sleep duration of 10 h or more and suicide attempt. The fifth limitation concerns the exploration of other psychiatric disorders using validated questionnaires instead of standard clinical assessment and the inclusion of these components in statistical analyses. Recent work in a national adult cohort (National Epidemiologic Survey on Alcohol and Related Conditions) has shown that sleep disturbance is associated with an increased risk of suicide attempt independent of psychopathology^39^. In addition, a longitudinal study of youth at high risk for suicide observed that objectively and subjectively measured sleep disturbance predicted increased acute suicidal ideation in this population, independent of depressed mood^77^.

The study of sleep disorders has many advantages especially because of the trans-nosographic nature of sleep disorders^78^. Indeed, sleep disorders may be isolated or associated with mental health or psychiatric disorders^79,80^. They are also very sensitive and vary in the short and long term^16^. Finally, sleep is a cross-sectional and non-stigmatizing component and therefore accessible for evaluation by all health professionals. The study of sleep in suicidology should be a key intervention target because sleep can easily be assessed using simple screening and diagnostic tools^16^, and effective treatments exist to improve sleep—these treatments could thus participate in decreasing suicidal risk in adolescents^81,82^, as highlighted by several recommendations^42,83,84^. However, current treatment after a suicidal act is mainly focused on the management of an associated psychological or psychiatric disorder including addictions (psychotherapy and/or pharmacotherapy) as well as on the environment of the young person (family therapy, school intervention, etc.), but also on the development of protective factors, particularly via 'recontact devices'^85^. In practice, these treatments do usually not include sleep interventions or with non-optimal methodology. However, a recent meta-analysis shows that Cognitive-Behavioral Therapy (CBT) targeting insomnia (CBT-insomnia) for adolescents presents beneficial effects on sleep latency, which we have seen is strongly increased in suicidal individuals, and on sleep efficiency^86^. In adults, an improvement of sleep by CBT-i could have a major impact on suicidal risk, either for sleep disorders are associated with a psychological disorders or not^87^. Thus, there is a clear need for high-quality clinical trials evaluating the efficacy of sleep-targeted interventions (psychoeducation, CBT-insomnia, etc.) in reducing relapse to suicide attempts in adolescents. Future studies using objective measures of sleep disturbances are needed to help identify them, and to clarify the link between sleep disturbance and suicide. For greater validity, these measures should be performed under ecological conditions, i.e., in the adolescent's usual home and school environment. For this purpose, actimetry would allow to explore sleep in a more sensitive way^88^.

## Conclusion

Major differences were observed between the sleep of adolescents in the 4 weeks prior to a suicide attempt and the sleep of non-suicidal adolescents, with a shorter total sleep duration and longer sleep latency on school days. Reduced sleep duration was associated with poorer psychological well-being, as well as with school-related difficulties. Our results suggest the potential value of including sleep assessment in the clinical assessment of suicide risk, and that these symptoms may be used as a potential biomarker for risk of suicidal behavior as part of efforts in suicide prevention and adolescent mental health. In the current context, increased attention to adolescent sleep is warranted, whether in the case of suicidal ideation, psychological difficulties (particularly since the Covid-19 pandemic) or in contexts of increased screen use in the evening. In addition, a systematic assessment of the suicidal risk in young people with sleep disorders would be relevant. Future suicide prevention and mental health intervention programs should incorporate more targeted sleep assessments and interventions.

## Data Availability

Data are available and can be consulted and transmitted by contacting by e-mail Julie Rolling: julie.rolling@chru-strasbourg.fr.
